# Vegetable Activated Charcoal for Human Consumption Reduces Selected PFAS Levels in a Bile Secretion Model: Cues on Its Possible Clinical Use

**DOI:** 10.1021/acs.chemrestox.5c00510

**Published:** 2026-05-05

**Authors:** Alessandro Bonetto, Luca De Toni, Andrea Di Nisio, Laura Pagnin, Alberto Ferlin, Antonio Marcomini, Carlo Foresta

**Affiliations:** † DAIS-Department of Environmental Sciences, Informatics and Statistics, University Ca’ Foscari of Venezia, Venezia 30172, Italy; ‡ Department of Medicine, Unit of Andrology and Reproductive Medicine, University of Padova, Padova 35128, Italy; # Department of Psychology and Health Sciences, Pegaso University, Napoli 80143, Italy

## Abstract

Perfluoroalkyl substances (PFAS) are pollutants with relevant accumulation in humans, and the enterohepatic circulation of PFAS secreted in bile sustains their persistence. A significant increase in fecal excretion has been experimentally assessed with the use of oral adsorbents with negligible gut absorption. Here, we evaluated *in vitro* the use of activated charcoal (AC) for human consumption, as sorption material for a panel of PFAS, such as, perfluoro-butanoic acid (PFBA), perfluoro-butanesulfonic acid (PFBS), perfluoro-hexanoic acid (PFHxA), perfluoro-hexanesulfonic acid (PFHxS), perfluoro-octanoic acid (PFOA), and perfluoro-octanesulfonic acid (PFOS), in an experimental simulated bile juice (SBJ). The aim was to obtain preliminary data for possible clinical applications to reduce PFAS blood levels in humans. PFAS concentrations in experimental samples were quantified by liquid chromatography–mass spectrometry. In kinetic tests, equimolar solutions of single PFAS in SBJ were incubated with AC at 37 °C up to 120 min, and the time-dependent reduction of PFAS concentration was monitored. In thermodynamic tests, PFAS solutions in SBJ were incubated at increasing concentrations with AC for 24 h at 37 °C and the concentrations at equilibrium evaluated. Results were finally fitted with available models in order to characterize the PFAS interaction with AC. All PFAS showed more than 80% sorption on activated charcoal from simulated bile juice within 120 min. This suggests rapid and nearly complete removal. Modeling analysis indicated that the pseudo-first-order kinetic model best described short-chain PFAS, while PFOS and PFOA fitted better with the Elovich model. Thermodynamic analysis showed a general fitting with the Freundlich model, presumptive of a heterogeneous binding model. PFOS binding was concentration-dependent and was better described by the Sips model. These data are suggestive of a potential noninvasive intervention strategy to increase fecal PFAS excretion through the dietary use of AC, in order to mitigate health issues associated with PFAS exposure.

## Graphical abstract

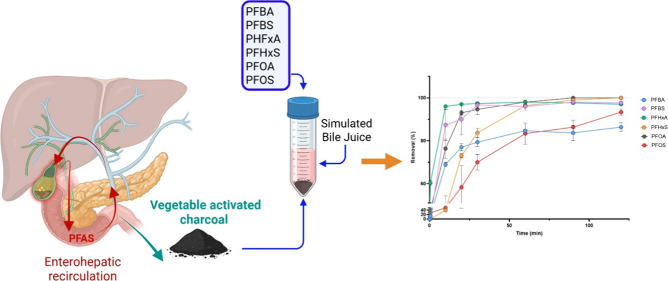

## Introduction

Perfluoroalkyl substances (PFAS) are anthropogenic chemicals with a partially (poly) or fully (per) fluorinated alkyl chain. Food and drinking water are primary sources of human exposure, though other environmental routes (e.g., inhalation) can contribute. Serum PFAS levels, recognized as exposure markers, are typically higher in males than females.^1–4^ The estimated plasma half-lives of two long-chain legacy compounds, perfluoro-octanesulfonic acid (PFOS) and perfluoro-octanoic acid (PFOA), are 5.4 and 2.3–3.8 years, respectively.^4^ Although much of the PFAS burden is protein-bound in plasma, relevant accumulation occurs in bile, skeletal muscle, liver, and kidneys.^1,5^ Tissue accumulation is thought to be sustained by enterohepatic circulation (EHC), being actively secreted in bile and subsequently reabsorbed in the gut with minimal fecal elimination.^6,7^

No therapeutic protocols are currently approved to reduce PFAS blood levels in humans. Experimental strategies have included long-term phlebotomy and apheresis.^8,9^ Alternative approaches have taken advantage of EHC to promote PFAS removal using oral adsorbents with negligible gut absorption. In murine models, the bile acid sequestrant cholestyramine enhanced fecal excretion of PFAS.^10^ Case reports in humans confirmed that cholestyramine (4 g three times daily for 28 weeks) significantly reduced circulating PFOS, PFOA, and PFHxS.^9,11^ However, the standard 12 g/day dosage is poorly tolerated, with high rates of constipation and therapy discontinuation.^12^ The use of colesevelam, a more recent and better tolerated bile acid sequestrant, showed a clear secretory effect on PFHxS and PFOS but a weak and less consistent impact on PFOA.^10^

Activated charcoal is a recognized treatment for PFAS removal from drinking water.^13–16^ In human adults, the oral use of vegetable activated charcoal (AC) is approved for acute drug intoxication, at a typical dose of 30–50 g as a gastric bolus. AC is also indicated in subchronic conditions, such as bloating and uremic pruritus, at an overall daily dose of 6 g for up to 6 weeks.^17,18^ Taken together, this evidence suggests the potential of oral AC to reduce elevated PFAS levels in highly exposed individuals, possibly in combination with a choleretic substance in order to stimulate bile secretion. In this study, we evaluated the possible use of AC approved for human consumption as sorption material for selected long-chain and short-chain PFAS in an experimental bile secretion model, providing preliminary cues on its possible clinical application.

## Experimental Procedures

### Chemicals

Vegetable activated charcoal for use in food (cat# 0189–1000-g), produced by coconut combustion and activated with water steam, was purchased from Galeno (Carmignano, Prato, Italy). The reported technical data of AC were as follows: average grain size of 15–35 μm, with 90% less than 74 μm, iodine number of approximately 1000 mg/g, and average surface area of 1800 m^2^/g. Perfluoro-butanoic acid (PFBA, 98%, cat. no. 164194–25G), perfluoro-butanesulfonic acid (PFBS, 97%, cat. no. 562629–25G), perfluoro-hexanoic acid (PFHxA, 97%, cat#29226–5 ML), potassium perfluoro-hexanesulfonate (PFHxK, cat. no. 88818–10MG), heptadecafluoro-octanesulfonic acid potassium salt (PFOK, cat. no. 95181–25MG), and PFOA (95%, cat. no. 17148–25g) were purchased from Merck-Sigma (Milano, Italy). Mass-labeled PFAS Mixture/Solution (cat# MPFAC-24ES) was purcahed from Wellington Laboratories (Southgate, Ontario, Canada). Simulated Bile Juice (SBJ, BZ263, Biochemazone, Alberta, Canada) was used as a bile secretion model, given the specific qualitative composition including both organic and inorganic ingredients, as per the certificate of analysis.

### Experimental Conditions

Experimental conditions were defined according to an estimated overall daily oral dose of AC of 7 g/day, fractionated into two single doses of 3.5 g each, to be taken away from meals. Considering a daily production of bile secretion of approximately 600 mL/day divided into four fractions, each of 150 mL, the resulting carbon/bile secretion ratio was 23.3 mg/mL. For operational simplicity, in order to reduce weighing errors due to the low handling of the material, a solid/liquid ratio of 30 mg/mL was considered.

Using a reference test concentration of PFOA of 100 ng/mL, the same molar test concentration was used for each individual selected PFAS (Table S1). Operationally, each PFAS was weighed into a 50 mL polypropylene (PP) test tube, dissolved with 50 mL of a 9:1 ultrapure water:methanol (UPW:MeOH) solution and named Stock1 solution. The Stock1 solutions of each PFAS were then appropriately diluted with SBJ, obtaining 50 mL of an intermediate Working1 solution at an individual concentration of 604 nmol/L.

Test samples were obtained by weighing 750 mg of activated carbon in a 50 mL PP tube to which 15 mL of SBJ was added and let to equilibrate for 12 h at 37 °C under orbital shaking at 250 rotations per minute (rpm).

In kinetic tests, 10 mL of Working1 solutions of each PFAS were added into equilibrated AC/SBJ suspension, obtaining the same test concentration of 241 nmol/L, compatible with the highest mean plasma levels reported long-chain PFAS.^1,10^ Each sample was homogenized by vortexing for 5 s and then let to incubate at 37 °C under orbital shaking at 250 rpm up to 120 min. Each PFAS was tested in triplicate. Control sample (CTRL), in which AC was omitted, was tested in singleton based on the precision and consistency of the method. Test samples underwent consecutive withdrawals of 250 μL aliquots immediately after adding Working1 solutions and after 0.5, 10, 20, 30, 60, 90, and 120 min. Aliquots were immediately collected, filtered with 0.45 μm regenerated cellulose filters (Claristep, Sartorius, Gottinga, Germany), and collected into 0.3 μL PP vials. After filtration, 5 μL of each aliquot was diluted 100-fold with the diluent solution to a final volume of 500 μL. 10 μL portions of internal standards (IS) were then added to each diluted aliquot and stored at 4 °C until analysis.

In thermodynamic tests, 300 mg of AC was dispersed in 10 mL of SBJ in a 15 mL polypropylene (PP) test tube, and appropriate amounts of the Working1 solutions were added. Samples were maintained for 24 h at 37 °C under constant shaking at 250 rpm by an orbital shaker. Finally, samples were centrifuged at 11,139*g* (Eppendorf Centrifuge 5910 Ri) and 5 μL of supernatant was diluted 100-fold with the diluent solution to a final volume of 500 μL in 0.7 mL poly propylene vials. 10 μL of internal standard was then added to the diluted samples and then stored at 4 °C until quantitative analysis.

### Liquid Chromatography–Mass Spectrometry

Quantitative analysis was performed with a liquid chromatograph coupled to a mass detector (LC-MS/MS TQ Absolute, Waters). The chromatographic separation was performed using a 1.7 μm, 2.1 × 50 mm BEH C18 column, and the mobile phases were 2 mM NH4Ac in UPW and 2 mM in NH4Ac MeOH:AcCN 1:1 mixuture. The method involved the quantification of PFAS through correction with a labeled IS and using a 7-point calibration curve (Table S2). Vials were maintained at 10 °C for the entire duration of the analysis, using 50 μL for each run. Table S3 shows the mass spectrometer conditions. The analysis was conducted in negative mode, with a source temperature of 100 °C, desolvation temperature of 350 °C, desolvation flow rate of 900 L/h, and gas cone of 150 L/h. Method validation, by replicate analyses on spiked samples across multiple concentration levels, showed a relative standard deviation (RSD) ranged between 2 and 8%, demonstrating precision and consistency.

### Modeling Analysis

In kinetic studies, in order to characterize the nature of interactions between PFAS and AC in bile simulants and to address the possible influence of PFAS chain length on the adsorption rate, data were fitted with pseudo-first-order (Lagergren), pseudo-second-order (Ho), and Elovich kinetic models. Applied models were as follows:

#### Lagergren or Pseudo-First Order (pFO)

$$\ln\left(q_{e}-q_{t}\right)=\ln q_{e}-k_{1}t$$

where: *q*_*e*_ is the molar concentration adsorbed at equilibrium; *q*_*t*_ is the molar concentration adsorbed at time *t*; and *k*_1_ is the kinetic constant.

#### Ho Equation or Pseudo-Second Order (pSO)

$$\frac{t}{q_{t}}=\frac{1}{\left(k_{2}{q_{e}}^{2}\right)}+\frac{t}{q_{e}}$$

where *q*_*e*_ is the molar concentration adsorbed at equilibrium; *q*_*t*_ is the molar concentration adsorbed at time *t*; and *k*_2_ is the kinetic constant.

#### Elovich Equation

$$q_{t}=\frac{1}{\beta }\ln\left(1+\alpha \beta t\right)$$

where *q*_*t*_ is the molar concentration adsorbed at time *t*; α is the initial absorpion rate; and β is the parameter is related to the heterogeneity of the adsorption sites.

In thermodynamic studies, in order to evaluate the role of sorbent saturation and to address the mechanism of adsorption mechanism (e.g., chemo-adsorption *vs* physi-adsorption), a fitting analysis with classical thermodynamic models, namely, the Langmuir isotherm, Freundlich isotherm, Temkin isotherm, and Sips model, was performed. The reference models were as follows:

#### Langmuir Isotherm

$$q_{e}=\frac{q_{\max}K_{L}C_{e}}{1+K_{L}C_{e}}$$

where *q*_*e*_ is the molar concentration adsorbed at equilibrium (mol/g); *C*_*e*_ is the molar concentration at equilibrium (mol/L); *q*_max_ is the maximum adsorption capacity (mol/g); and *K*_*L*_ is the Langmuir constant.

#### Freundlich Isotherm

$$q_{e}=K_{F}C_{e}^{1/n}$$

where *q_e_* is the molar concentration adsorbed at equilibrium (mol/g); *C_e_* is the molar concentration at equilibrium (mol/L); *n* is the surface heterogeneity; and *K_F_* is the Freundlich constant.

#### Temkin Isotherm

$$q_{e}=\frac{RT}{b_{T}}\ln\left(A_{T}C_{e}\right)$$

where *q*_*e*_ is the molar concentration adsorbed at equilibrium (mol/g); *C*_*e*_ is the molar concentration at equilibrium (mol/L); *b*_*T*_ is the adsorption energy (J/mol); *A*_*T*_ is the Temkin constant; *R* is the universal gas constant (J/mol × K); and *T* is the absolute temperature (K).

#### Sips Model

$$q_{e}=\frac{q_{\max}{\left(K_{S}C_{e}\right)}^{n}}{1+{\left(K_{S}C_{e}\right)}^{n}}$$

where *q*_*e*_ is the molar concentration adsorbed at equilibrium (mol/g); *C*_*e*_ is the molar concentration at equilibrium (mol/L); *q*_max_ is the maximum adsorption capacity (mol/g); *n* is the surface heterogeneity; and *K*_*S*_ is the Sips constants.

### Statistical Analysis

Statistical analysis was performed with GraphPad Prism 10.6.1 (GraphPad Software, Boston, Massachusetts USA). One-way analysis of variance (ANOVA) followed by Tukey’s post hoc test for multiple comparisons among time points was performed to assess the effect of contact time on the sorption kinetics of the selected PFAS.

## Results and Discussion

### Kinetic Tests

Preliminary experiments were performed throughout an overall observation time of 48 h, showing the achievement of a steady state adsorption rate of PFAS on AC within 120 min (Figure S1). Subsequent experiments then focused on this observation period, with more frequent sampling times in the first 60 min.

Results of PFAS quantification, distinguished by analyte and sampling time, are reported in Table 1. Importantly, PFAS content in naïve AC (CTRL-AC) was found at concentrations below the limit of detection (0.1 μg/L), with the exception of PFBA, PFHSX, and PFOA for which background values were, respectively, 2.81, 0.46, and 0.46 0.1 μg/L. This was likely attributable to the ubiquity of PFAS, appearing to be present in detectable concentrations in any matrix (e.g., surface water, drinking water). However, these background values represented less than 0.5% of the control values, allowing for the evaluation of 99.5% removals.

**Table 1:** Quantification of the Selected Perfluoroalkyl Substances (PFAS) in Kinetic Tests of Sorption on Vegetable Activated Charcoal (AC) for Human Consumption^b^

	PFAS (ng/mL)					
time (min)	PFBA	PFBS	PFHxA	PFHxS	PFOA	PFOS
CTRL	55.4 ± 0.4	96.2 ± 1.5	75.1 ± 0.3	139.2 ± 1.8	113.2 ± 3.1	150.0 ± 5.4
0.5	50.9 ± 1.3^a^	96.6 ± 6.9	29.9 ± 0.5^a^	143.0 ± 6.0	69.1 ± 6.2^a^	109.0 ± 16.1^a^
10	20.0 ± 0.5^a^	12.4 ± 7.2^a^	2.9 ± 0.1^a^	85.7 ± 2.5^a^	27.2 ± 4.7^a^	75.3 ± 6.4^a^
20	15.4 ± 0.9^a^	9.8 ± 7.3^a^	2.2 ± 0.2^a^	38.2 ± 1.0^a^	8.5 ± 0.9^a^	62.5 ± 15.5^a^
30	14.2 ± 1.3^a^	3.2 ± 1.3^a^	2.0 ± 0.1^a^	23.4 ± 2.6^a^	6.7 ± 2.8^a^	45.1 ± 5.3^a^
60	11.2 ± 0.3^a^	3.9 ± 1.9^a^	1.8 ± 0.0^a^	5.6 ± 0.6^a^	3.0 ± 0.7^a^	25.1 ± 6.9^a^
90	11.9 ± 2.1^a^	1.9 ± 0.1^a^	1.9 ± 0.4^a^	2.3 ± 0.2^a^	0.9 ± 0.02^a^	20.3 ± 5.0^a^
120	10.3 ± 1.0^a^	2.1 ± 0.4^a^	2.3 ± 0.2^a^	0.8 ± 0.2^a^	0.2 ± 0.03^a^	9.8 ± 1.8^a^
CTRL-AC	2.81 ± 1.1	<0.1	<0.1	0.46 ± 0.3	0.46 ± 0.4	<0.1

a Significance: *P* < 0.0001 vs respective CTRL.

b Abbreviations: PFBA, perfluoro-butanoic acid; PFBS, perfluoro-butanesulfonic acid; PHFxA perfluoro-hexanoic acid; PFHxS, perfluoro-hexanesulfonic acid; PFOA, perfluoro-octanoic acid; PFOS, perfluoro-octanesulfonic acid; CTRL, control sample in which AC was omitted, CTRL-AC, control sample of AC in which PFASs were omitted. All standard deviations refer to three independent experiments. Standard deviations of CTRL and CTRL-AC result from three consecutive assessments of the same sample.

In general, a discrepancy was observed between the concentration of individual PFAS at time *t* = 0 and that of the control sample in which AC was omitted. This suggests that, especially for highly hydrophobic carboxylic PFAS, such as the long-chain PFOA, despite sampling operations performed within 30 s from AC addition, the adsorption rate on AC was very high, resulting in a significant reduction of PFAS concentration. This evidence was observed for all of the long-chain PFAS evaluated. This is consistent with the octanol–water partition coefficients (log K_ow_) for the analyzed PFAS, which represents a good descriptor of PFAS partitioning in aqueous solution (Table S4). In general, it was observed that the higher this parameter, the greater was the reduction of PFAS concentration over the first 30 s of exposure to AC.

The percentage-removal profile for each individual PFAS by the AC, corrected for background values, is reported in Figure 1. Within 120 min, the removal profiles of PFBS, PFHxA, PFHxS, and PFOA were compatible with the achievement of a steady state condition, with stable removal percentages of ∼100%. A relevant deviation from this pattern was observed for PFOS, whose sorption kinetic appeared slower, achieving 83.3% after 1 h, compared to >95% for the aforementioned PFAS, despite the achievement of a final removal of >90% at 120 min. Differently, PFBA achieved a final removal rate of 86% at the end of the experiment.

**Figure 1:**
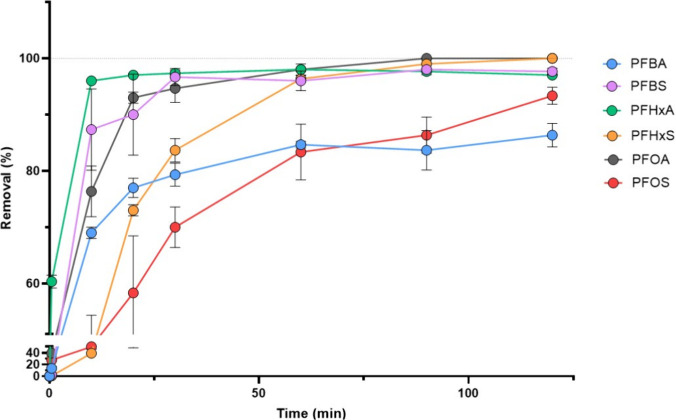
Experimental evaluation of the time-dependent removal of a panel of perfluoroalkyl substances from a simulated bile juice by activated charcoal for human consumption. Data are reported as the mean value ± standard deviation of a technical replicate. Abbreviations: PFBA, perfluoro-butanoic acid; PFBS, perfluoro-butanesulfonic acid; PHFxA perfluoro-hexanoic acid; PFHxS, perfluoro-hexanesulfonic acid; PFOA, perfluoro-octanoic acid; PFOS, perfluoro-octanesulfonic acid.

The data were fitted with pseudo-first-order (Lagergren), pseudo-second-order (Ho), and Elovich kinetic models in order to provide a kinetic model of interactions between PFAS and AC in bile secretion and to address the role of the carbon chain length on the initial adsorption rate. Results of kinetic parameter modeling are reported in Table 2. Within the limits of experimental variability, the adsorption of PFAS by AC in an aqueous environment were better described by a pSO model, especially for long-chain PFAS that better adapts to adsorption mechanisms involving chemical bonds or electron exchange, thus not only relying on physical surface forces. This model is characterized by a rapid initial adsorption rate due to the large excess of free sites followed by a gradual approach to equilibrium. In particular, different from the other tested PFASs with shorter carbon chain, the Elovich model better described the kinetic profile of PFOA and PFOS.

**Table 2:** Analysis of Kinetic Model Fitness for Perfluoroalkyl Substance (PFAS) Absorption to Vegetable Activated Charcoal for Human Use (AC), According to Pseudo-First-Order (pFO), Pseudo-Second-Order (pSO), or Elovich Models^a^

PFAS	replicate	model	*R* ^2^	RMSE	*q*_e_	*k*_1_	*k*_2_	α	β
**PFBA**	#1	pFO	0.9866	3.05 × 10^–04^	0.0067	0.172			
		**pSO**	**0.9976**	**1.29 × 10^–04^**	**0.0071**		**44.2**		
		Elovich	0.9667	4.82 × 10^–04^				0.007954	890
	#2	pFO	0.9952	1.93 × 10^–04^	0.0069	0.153			
		**pSO**	**0.9973**	**1.45 × 10^–04^**	**0.0074**		**36.1**		
		Elovich	0.9550	5.88 × 10^–04^				0.006151	824
	#3	pFO	0.9972	1.41 × 10^–04^	0.0067	0.166			
		**pSO**	**0.9935**	**2.16 × 10^–04^**	**0.0071**		**45.0**		
		Elovich	0.9376	6.68 × 10^–04^				0.007634	892
**PFBS**	#1	**pFO**	**0.9953**	**2.99 × 10^–04^**	**0.0104**	**0.236**			
		PSO	0.9781	6.45 × 10^–04^	0.0111		34.4		
		Elovich	0.8954	1.41 × 10^–03^				0.012745	568
	#2	**pFO**	**0.9924**	**3.76 × 10^–04^**	**0.0103**	**0.233**			
		pSO	0.9762	6.68 × 10^–04^	0.0110		33.7		
		Elovich	0.8963	1.39 × 10^–03^				0.011909	567
	#3	**pFO**	**0.9752**	**7.11 × 10^–04^**	**0.0104**	**0.135**			
		pSO	0.9669	8.21 × 10^–04^	0.0113		18.5		
		Elovich	0.9117	1.34 × 10^–03^				0.005265	486
**PFHxA**	#1	**pFO**	**0.9998**	**3.86 × 10^–05^**	**0.0077**	**1.956**			
		pSO	0.9998	3.89 × 10^–05^	0.0078		413.9		
		Elovich	0.9739	4.21 × 10^–04^				25.43843	1889
	#2	**pFO**	**0.9998**	**3.95 × 10^–05^**	**0.0077**	**1.948**			
		pSO	0.9998	4.09 × 10^–05^	0.0078		411.4		
		Elovich	0.9735	4.24 × 10^–04^				24.3875	1883
	#3	**pFO**	**0.9999**	**2.56 × 10^–05^**	**0.0077**	**1.893**			
		pSO	0.9995	5.70 × 10^–05^	0.0078		394.5		
		Elovich	0.9702	4.50 × 10^–04^				18.86691	1849
**PFHxS**	#1	**pFO**	**0.9974**	**2.36 × 10^–04^**	**0.0115**	**0.058**			
		pSO	0.9904	4.49 × 10^–04^	0.0134		5.2		
		Elovich	0.9739	7.42 × 10^–04^				0.001854	352
	#2	**pFO**	**0.9931**	**3.87 × 10^–04^**	**0.0116**	**0.058**			
		pSO	0.9817	6.28 × 10^–04^	0.0135		5.1		
		Elovich	0.9616	9.11 × 10^–04^				0.001787	345
	#3	**pFO**	**0.9954**	**3.10 × 10^–04^**	**0.0116**	**0.058**			
		pSO	0.9864	5.36 × 10^–04^	0.0135		5.1		
		Elovich	0.9684	8.15 × 10^–04^				0.00186	351
**PFOA**	#1	pFO	0.9770	4.86 × 10^–04^	0.0083	1.348			
		pSO	0.9861	3.78 × 10^–04^	0.0085		201		
		**Elovich**	**0.9950**	**2.27 × 10^–04^**				**0.1053**	**945**
	#2	pFO	0.9131	1.00 × 10^–03^	0.0089	0.155			
		pSO	0.9685	6.03 × 10^–04^	0.0087		94.9		
		**Elovich**	**0.9845**	**4.23 × 10^–04^**				**0.0395**	**877**
	#3	pFO	0.9564	5.71 × 10^–04^	0.0087	1.044			
		pSO	0.9776	4.09 × 10^–04^	0.0089		121		
		**Elovich**	**0.9794**	**3.92 × 10^–04^**				**0.1009**	**984**
**PFOS**	#1	pFO	0.9561	6.69 × 10^–04^	0.0089	0.053			
		pSO	0.9705	5.48 × 10^–04^	0.0102		6.73		
		**Elovich**	**0.9843**	**4.00 × 10^–04^**				**0.002**	**534**
	#2	pFO	0.8649	1.12 × 10^–03^	0.0088	0.067			
		pSO	0.8910	1.01 × 10^–03^	0.0097		11.2		
		**Elovich**	**0.9546**	**6.48 × 10^–04^**				**0.012**	**785**
	#3	pFO	0.7904	1.31 × 10^–03^	0.0085	0.082			
		pSO	0.8271	1.19 × 10^–03^	0.0091		16.5		
		**Elovich**	**0.9525**	**6.25 × 10^–04^**				**0.037**	**968**

a Abbreviations: PFBA, perfluoro-butanoic acid; PFBS, perfluoro-butanesulfonic acid; PHFxA perfluoro-hexanoic acid; PFHxS, perfluoro-hexanesulfonic acid; PFOA, perfluoro-octanoic acid; PFOS, perfluoro-octanesulfonic acid; *R* ^2^, determination coefficient; RMSE, root-mean-square error; *q*_e_, molar concentration adsorbed at equilibrium; *k*_1_, pFO kinetic constant; *k*_2_, pSO kinetic constant; α, initial absorpion rate; β, parameter related to the heterogeneity of the adsorption sites; Parameters of the best describing model for each replicate, according to the highest *R* ^2^ value and lowest RMSE value, are in bold.

### Thermodynamic Tests

Raw data of concentration at equilibrium (*C_e_*) *vs* initial concentration (*C*_0_) curves for each tested PFAS are reported in Figure S2. In qualitative terms, long-chain PFAS, namely, PFOA and PFOS, showed a nearly linear increase of *C_e_* for low *C*_0_ values followed by a nearly exponential surge. On the other hand, short-chain PFAS showed a nearly linear increase of *C_e_* throughout the whole range of *C*_0_.

Results of fitting analysis on thermodynamic models are reported in Table 3 and corresponding graphical representations are reported in Supporting Information, Figure S3. Several models have been adopted, whose detailed description has been recently reviewed by Mozaffari Majd et al.^19^ Briefly, the Langmuir isotherm in its linear form relies on the homogeneous surface of the adsorbent material, with energetically equivalent sites forming a single layer of sorped molecules with negligible mutual interaction. The Freundlich model, on the other hand, assumes that the material surface is heterogeneous, with the sorption energy varying according to the active sites and the possible formation of a multilayer of adsorbed molecules. The Temkin equation is widely used to study the thermodynamic behavior, as it links the adsorption energy to the surface coverage. However, this relationship is less reliable when saturation occurs at high concentrations. Finally, the Sips equation describes intermediate situations, describing Langmuir-type behavior at high concentrations and Freundlich-type behavior at low concentrations and considering the heterogeneity of the active sites. The sorption of short-chain C4 PFAS (PFBA, PFBS) is best described by the Freundlich model, showing a nearly linear dependence of the adsorbed amount on the residual concentration (*C_e_*) associated with low levels of saturation of active sites or low binding energy. This evidence is consistent with the chemical–physical characteristics of short-chain PFAS, which, compared to their long-chain counterparts, have lower octanol–water partition coefficients (Table S3). The highest values of *K_L_* have been observed for long-chain PFAS, confirming that the chain length is an important parameter in the adsorption of PFAS in carbon matrices such as activated carbon. However, chain length might affect the steric hindrance of the molecules, hampering the diffusion and adsorption into very narrow pores, thus also limiting their maximum adsorption capacity. This hypothesis is confirmed by the calculated *q*_max_ value of PFAS and PFOA, by the SIPS model, which is lower than that of PFBA.

**Table 3:** Analysis of Thermodynamic Model Fitness for Perfluoroalkyl Substance (PFAS) Absorption to Vegetable Activated Charcoal for Human Use (AC), According to the Langmuir Isotherm, Freundlich Isotherm, Temkin Isotherm, or Sips Model^a^

PFAS	model	***R*** ^2^	RMSE	*q*_max_	*K*_L_	*K*_F_	*n*	*A*_T_	*b*_T_	*K*_S_
**PFBA**	Langmuir	0.974	0.005	44714	0.000					
	**Freundlich**	**0.984**	**0.004**			**2.187**	**0.871**			
	Temkin	0.951	0.008					74.9	42367	
	Sips	0.984	0.004	0.495			1.32			8.77
**PFBS**	Langmuir	0.903	0.007	0.114	345					
	**Freundlich**	**0.948**	**0.005**			**0.268**	**3.787**			
	Temkin	0.941	0.006					5591	120617	
	Sips	0.948	0.005	1626			0.26			0.000
**PFHxA**	Langmuir	0.824	0.012	27770	0.000					
	**Freundlich**	**0.966**	**0.005**			**34.00**	**0.474**			
	Temkin	0.627	0.018					121	66348	
	Sips	0.966	0.005	4102			2.11			0.008
**PFHxS**	Langmuir	0.818	0.005	0.057	101					
	**Freundlich**	**0.936**	**0.003**			**0.159**	**2.670**			
	Temkin	0.726	0.007					38098	453693	
	Sips	0.936	0.003	5843			0.37			0.000
**PFOA**	Langmuir	0.953	0.006	0.112	79					
	Freundlich	0.872	0.010			0.323	2.37			
	Temkin	0.850	0.010					1934	141784	
	**Sips**	**0.962**	**0.005**	**0.098**			**1.37**			**571**
**PFOS**	Langmuir	0.955	0.003	0.088	141					
	Freundlich	0.844	0.006			0.191	3.44			
	Temkin	0.907	0.004					1601	135889	
	**Sips**	**0.976**	**0.002**	**0.079**			**1.47**			**1781**

a Abbreviations: PFBA, perfluoro-butanoic acid; PFBS, perfluoro-butanesulfonic acid; PHFxA perfluoro-hexanoic acid; PFHxS, perfluoro-hexanesulfonic acid; PFOA, perfluoro-octanoic acid; PFOS, perfluoro-octanesulfonic acid; *R* ^2^, determination coefficient; RMSE, root-mean-square error; *q*_max_, molar concentration adsorbed at equilibrium; *K_L_*, Langmuir constant; *K_F_*, Freundlich constant; *n*, surface heterogeneity; *A_T_*, Temkin constant; *b_T_*, adsorption energy; *K_S_*, Sips constants. Parameters of the best describing model, according to the highest value of *R* ^2^ and the lowest value of RMSE for each replicate, are in bold.

To the best of our knowledge, this is the first study providing evidence for a significant sorption of legacy PFAS of activated charcoal from a human bile secretion model. Considering the complex composition of bile, including bile acids, electrolytes, and organic metabolites that can compete for the absorption to AC, the observed net removal of at least 80% of the compounds sustains the possible tole of AC to reduce the pool of PFAS susceptible to enterohepatic recirculation, thus promoting their fecal elimination.^10^

At first, the kinetic and thermodynamic adsorption of a PFAS panel on a commercially available AC for oral use were characterized from a bile secretion model. Importantly, the experimental conditions were adjusted to match the approved daily oral dose of AC for the reduction of excessive intestinal gas accumulation and bloating.^20^ Our results show that incubating with AC is linked to an average decrease in PFAS levels by more than 80% through adsorption, which generally reaches equilibrium within 2 h. For medium- to short-chain PFAS, the adsorption follows the Freundlich model, indicated by a heterogeneous binding mode based on the surface sorption energy, availability of active sites, and possible multilayer formation. Differently, long-chain PFAS are better described by the Sips model, relying on a homogeneous or heterogeneous binding mode according to the concentation of the sorped compound.^19^ These findings are consistent with recent work by Abulikemu et al., who found that perfluoroalkyl carboxylic acids fit well with the Freundlich model, whereas PFOS deviated from this pattern depending on the granulometry of the adsorbent material.^21^ Interestingly, previous data from Abulikemu showed the achievement the sorption equilibrium between 6 and 18 h for PFHxS, different from the 2 h observed in the current study.^21^ This discrepancy might be due to the execution of all experiments at 37 °C, which is largely compatible with a rate-doubling compared to room temperature.

Of note, the maximum adsorption capacities of AC measured in the present study are approximately 100 times lower compared to those reported for other aqueous matrices.^22^ This difference can be attributed not only to the limitations of the test, since real conditions were simulated without bringing the material to saturation, but more importantly to the different composition of natural aqueous matrices compared to the BJC.^23^ In fact, compared to the 0.05–0.2% solute content of natural mineral waters, essentially constituted of inorganic salts, bile secretion typically shows a 5% content of organic solutes including 61% bile acids surfactants, 12% fatty acids, and 9% cholesterol.^23,24^ Accordingly, it can be speculated that, given the amphiphilic nature of the abovementioned organic compounds, competition/interference processes may have been established with PFAS at the surface binding to the AC, thus generating possible deviations from the models typically considered as reliable.^25^ To this regard, sorption kinetics for PFOA and PFOS on AC are better described by the Elovich model, different from the other tested PFAS. Data from Militao et al., using rice straw-derived biochar, showed a pSO-like fitting for PFOS adsorption in purified water.^26^ Differently, the Elovich model was the best fitting model of lead ion adsorption on coconut AC as described by Largitte and Pasquier.^27^ It can thus be essentially speculated the possible modification of the hydrophilic–lipophilic balance of long-chain PFAS by bile salts, being more hydrophobic and susceptible to the surfactant activity of the latter, resulting in the first binding to the lower energy surface sites of the carbon saturate followed by the shifts to the higher energy surface sites, resulting in a decrease of the sorption rate.^27^ Nonetheless, our results suggest that the use of vegetable carbon associates with an average adsorption efficiency greater than 80% for all PFAS, even at the mg/L range reported for bile secretion.^5–7^ As a limitation, we acknowledge that the potential modification of AC sorbent activity by the exposure to the sequential gastric and duodenal fluid environment remains to be addressed. In addition, given the rather nonspecific adsorbent activity of AC, careful consideration of appropriate dosing is necessary to minimize possible nutrient malabsorption.^28^

Our preliminary findings indicate the possibility of a noninvasive pharmacological intervention to enhance fecal excretion of PFAS, particularly following the reduction or cessation of exposure to these substances. Accordingly, the dietary supplementation of AC may represent a minimally invasive approach to mitigate health risks associated with PFAS exposure. Importantly, access to an effective pharmacological method for lowering circulating PFAS levels is critical not only for communities subjected to significant contamination but also for the general population experiencing routine background exposure from everyday products containing PFAS.^29^ Further research is warranted to address the possible role of AC treatment in reducing the blood levels of PFAS.

## Supplementary Material



## Notes

Supplemental Table S1: Working concentrations of PFAS solutions; Supplemental Table S2: Recipe for PFAS calibration curve standards solutions; Supplemental Table S3: Parameters of LC-MS/MS analysis of PFAS; Supplemental Table S4: Molecular descriptors of tested PFAS; Supplemental Figure S1: 0–48 h analysis of PFOA removal from simulated bile juice by activated charcoal; Supplemental Figure S2: Row isothermal adsorption curves of PFAS on activated charcoal; Supplemental Figure S3: Fitting analysis curves of isothermal adsorption of PFAS on activated charcoal (PDF)
