# Supplementary material for: Vegetable Activated Charcoal for Human Consumption Reduces Selected PFAS Levels in a Bile Secretion Model: Cues on Its Possible Clinical Use
Source: Chem Res Toxicol. 2026 May 5;39(5):900–7. doi: 10.1021/acs.chemrestox.5c00510 (PMC13188163; doi:10.1021/acs.chemrestox.5c00510)
Supplement: Supplementary file 1 [file tx5c00510_si_001.pdf]

SUPPORTING INFORMATION

Vegetable activated charcoal for human consumption reduces selected PFAS levels in a bile secretion model: cues on its possible clinical use.

Alessandro Bonetto[†], Luca De Toni[‡], Andrea Di Nisio[#], Laura Pagnin[†], Alberto Ferlin[‡], Antonio Marcomini[†]*, Carlo Foresta[‡].
Equal contribution of the first two authors

Affiliations

[†] DAIS-Department of Environmental Sciences, Informatics and Statistics, University Ca' Foscari of Venezia, 30172 Venezia, Italy

[‡] Department of Medicine, Unit of Andrology and Reproductive Medicine, University of Padova, 35128 Padova Italy

[#] Department of Psychology and Health Sciences, Pegaso University, 80143 Napoli, Italy.

*** Correspondence**

Prof. Antonio Marcomini, DAIS-Department of Environmental Sciences, Informatics and Statistics, University Ca' Foscari of Venezia, Venezia, Italy. E-mail: marcomini@unive.it

Supplemental Table S1	Page 2
Supplemental Table S2	Page 3
Supplemental Table S3	Page 4
Supplemental Table S4	Page 5
Supplemental Figure S1	Page 6
Supplemental Figure S2	Page 7
Supplemental Figure S3	Page 8

Supplemental Table S1. Gravimetric and molar concentrations of perfluoro-alkys substances (PFAS) test solutions used for experimental absorption on activated charcoal.

PFAS	Gravimetric Concentration (ng/mL)	Molar Concentration (nmol/L)
Perfluoro-butanoic acid (PFBA)	52	241
Perfluoro-butanesulphonic acid (PFBS)	72	
Perfluoro-hexanoic acid (PFHxA)	76	
perfluoro-hexanesulfonic acid (PFHxS)	97	
Perfluoro-octanoic acid (PFOA)	100	
Perfluoro-octansulphonic acid (PFOS)	121	

Supplemental Table S2.
Recipe for calibration curve standards solutions (STD), used for the quantification of perfluoroalkyl-substances (PFAS), obtained by diluting 604 nmol/L Working 1 solution, together with internal standard (IS), with simulated bile juice (SBJ)

Labelling	Final Concentration (ng/L)	Working 1 volume (μL)	SBJ (μL)	IS (μL)
**STD1**	1	5	4995	10
**STD2**	5	25	4975	10
**STD3**	10	50	4950	10
**STD4**	50	250	4750	10
**STD5**	100	500	4500	10
**STD6**	500	2500	2500	10
**STD7**	1000	5000	-	10

Supplemental Table S3: Parameters used for perfluoroalkyl-substances (PFAS) analysis via liquid chromatography/mass spectrometry (LC MS/MS).

PFAS		Precursor (m/z)	Product (m/z)	Cone voltage (V)	Collision energy (V)	Quan	Retention Time (min)
Perfluoro-butanoic acid [Internal Standard]	PFBA	213	169	10	10	QUANT	2.3
	[^{13}C-PFBA]	217	172	10	10	QUANT	2.3
Perfluoro-butanesulphonic acid [Internal Standard]	PFBS	299	80<br>99	15<br>15	32<br>26	QUANT<br>QUAL	3.78
	[$^{13}C_3$-PFBS]	302	80<br>99	10<br>10	30<br>25	QUANT<br>QUAL	3.78
Perfluoro-hexanoic acid [Internal Standard]	PFHxA	313	119<br>269	5<br>5	22<br>10	QUAL<br>QUANT	4.11
	[$^{13}C_5$-PFHxA]	318	172<br>376	10<br>10	20<br>10	QUAL<br>QUANT	4.11
perfluoro-hexanesulfonic acid [Internal Standard]	PFHxS	399	80<br>99	10<br>10	35<br>30	QUANT<br>QUAL	5.14
	[$^{13}C_3$-PFHxS]	402	80<br>99	10<br>10	40<br>35	QUANT<br>QUAL	5.14
Perfluoro-octanoic acid [Internal Standard]	PFOA	413	169<br>369	10<br>10	15<br>10	QUANT<br>QUAL	5.36
	[$^{13}C_8$-PFOA]	421	172<br>376	5<br>5	15<br>10	QUAL<br>QUANT	5.36
Perfluoro-octansulphonic acid [Internal Standard]	PFOS	499	80<br>99	10<br>10	30<br>30	QUANT<br>QUAL	6.16
	[$^{13}C_8$-PFOS]	507	80<br>99	15<br>15	40<br>40	QUANT<br>QUAL	6.16

The QUANT/QUAL indexing of the Quan parameter describes the use of the indicated signal for, respectively, qualitative identification of the compound or quantitative analysis purposes

Supplemental Table S4. Hydrophobicity descriptors of perflouro-alkyl substances (PFAS) tested for sorption on vegetable activated charcoal (AC).

	**PFBA (C_4)**	**PFBS (C_4)**	**PFHxA (C_6)**	**PFHxS (C_6)**	**PFOA (C_8)**	**PFOS (C_8)**
Log K_{ow}	2.2#	2.3#	3.6#	3.7#	4.9#	5.0#
pK_a	0.08*	0.14*	-0.16*	0.14*	0.5*	0.14*
% $C_{t=0.5}/C_{Control}$	92	100	40	103	56	73

Abbreviations: Log K_{ow}, Octanol/Water Partition Coefficient- decimal logarithm; pKa, acid dissociation constant-p notation; % $C_{t=0.5}/C_{Controllo}$, percentage ratio between PFAS concentration after 0.5 minutes of exposure to AC and the control sample lacking AC; PFBA, perfluoro-butanoic acid; PFBS, perfluoro-butanesulphonic acid; PHFxA perfluoro-hexanoic acid; PFHxS, perfluoro-hexanesulfonic acid; PFOA, perfluoro-octanoic acid; PFOS, perfluoro-octanesulfonic acid; C_n, number of carbon atoms in the linear chain of the molecule.

*: data were retrieved from https://www.ncbi.nlm.nih.gov/books/NBK592145/table/ch4.tab2/

#: data were retrieved from PubChem

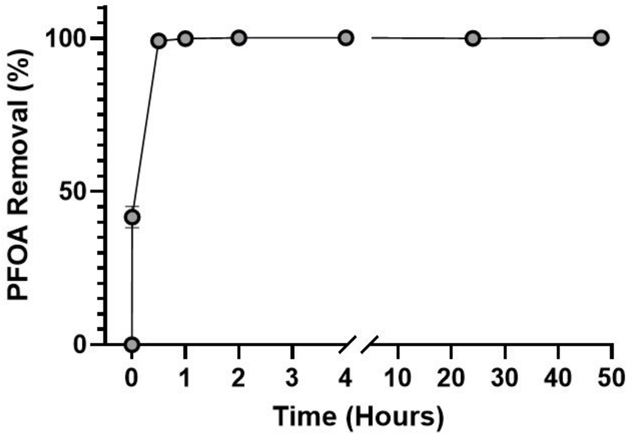

Supplemental Figure S1

Supplemental Figure S1

Experimental evaluation of the time-dependent removal of a panel of perfluoro-octanoic acid (PFOA), from a simulated bile juice, by activated charcoal for human consumption. Data are reported as the mean value ± standard deviation of a technical replicate.

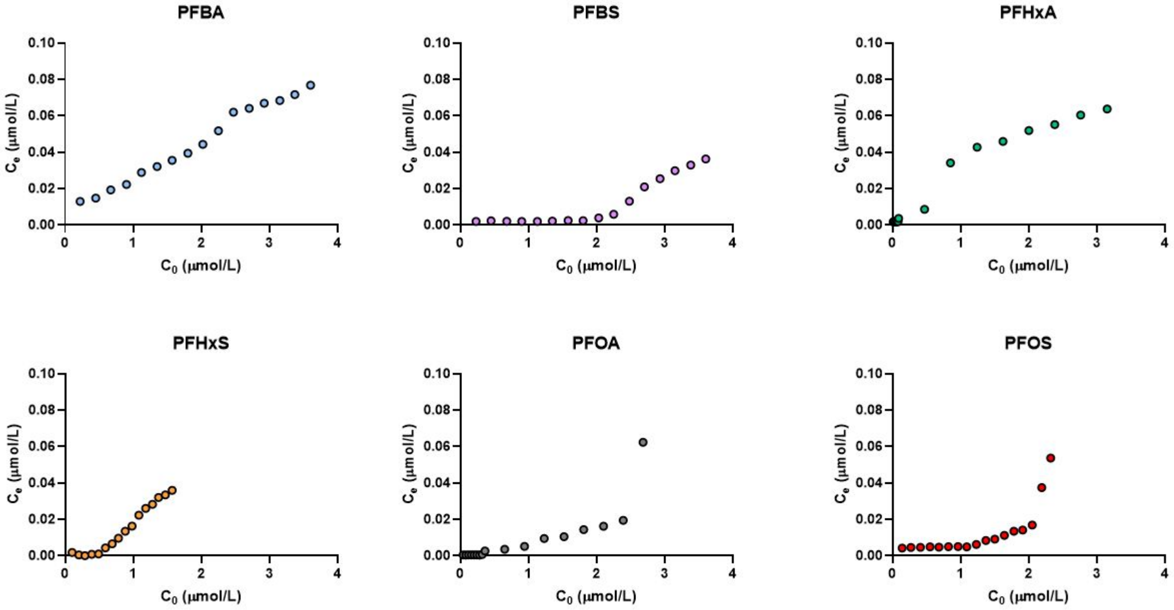

Supplemental Figure S2

Supplemental Figure S2

Row data of concentration at equilibrium (C_e) *vs* initial concentration (C_0) curves in isothermal adsorption assessment of a panel of perfluoro-alkyl substances, diluted in simulated bile juice, on activated charcoal for human consumption. Abbreviations: PFBA, perfluoro-butanoic acid; PFBS, perfluoro-butanesulphonic acid; PHFxA perfluoro-hexanoic acid; PFHxS, perfluoro-hexanesulfonic acid; PFOA, perfluoro-octanoic acid; PFOS, perfluoro-octanesulfonic acid.

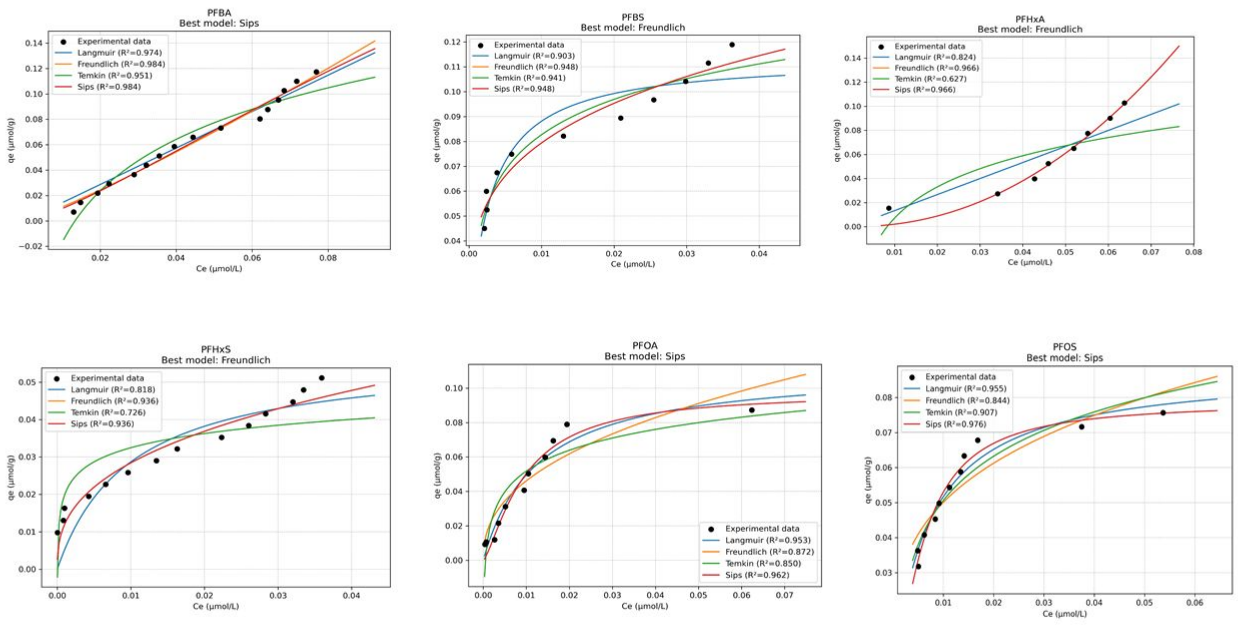

Supplemental Figure S3

Supplemental Figure S3

Graphical representation of the fitting analysis on isothermal adsorption assessment of a panel of perfluoro-alkyl substances, diluted in simulated bile juice, on activated charcoal for human consumption. Estimated concentration at equilibrium (q_e) *vs* initial concentration (C_0) curves according to the four models evaluated, Langmuir, Freundlich, Temkin and Sips, are reported with the corresponding determination coefficient (R^2). The Freundlich model curve for PFHxS, PFBS, and PFHxA is apparently missing because of the overlapping with the Sips curve.
